# Examining the Impact of Squaric Acid as a Crosslinking Agent on the Properties of Chitosan-Based Films

**DOI:** 10.3390/ijms22073329

**Published:** 2021-03-24

**Authors:** Ewa Olewnik-Kruszkowska, Magdalena Gierszewska, Sylwia Grabska-Zielińska, Joanna Skopińska-Wiśniewska, Ewelina Jakubowska

**Affiliations:** 1 Department of Physical Chemistry and Physicochemistry of Polymers, Faculty of Chemistry, Nicolaus Copernicus University in Toruń, Gagarina 7, 87-100 Toruń, Poland; sylwia.gz@umk.pl (S.G.-Z.); ewelina@doktorant.umk.pl (E.J.); 2 Department of Chemistry of Biomaterials and Cosmetics, Faculty of Chemistry, Nicolaus Copernicus University in Toruń, Gagarina 7, 87-100 Toruń, Poland; joanna@umk.pl

**Keywords:** chitosan, squaric acid, crosslinking, swelling, mechanical properties

## Abstract

Hydrogels based on chitosan are very versatile materials which can be used for tissue engineering as well as in controlled drug delivery systems. One of the methods for obtaining a chitosan-based hydrogel is crosslinking by applying different components. The objective of the present study was to obtain a series of new crosslinked chitosan-based films by means of solvent casting method. Squaric acid—3,4-dihydroxy-3-cyclobutene-1,2-dione—was used as a safe crosslinking agent. The effect of the squaric acid on the structural, mechanical, thermal, and swelling properties of the formed films was determined. It was established that the addition of the squaric acid significantly improved Young’s modulus, tensile strength, and thermal stability of the obtained materials. Moreover, it should be stressed that the samples consisting of chitosan and squaric acid were characterized by a higher swelling than pure chitosan. The detailed characterization proved that squaric acid could be used as a new effective crosslinking agent.

## 1. Introduction

Chitosan is a natural polymer consisting of randomly distributed β-(1-4)-linked D-glucosamine and N-acetyl-D-glucosamine units. The increasing interest in this polymer is related to its low toxicity and biocompatibility, as well as its susceptibility to enzymatic degradation. Moreover, contrary to a synthetic polymer, chitosan does not present immunogenicity. For this reason, chitosan is one of the polymers used for the formation of hydrogels, which have to meet a significant number of requirements set forth by the medical, pharmaceutical, and food packaging sectors. It is well known that hydrogels have to be characterized by improved mechanical properties, and that they have to ensure effective stability in different pH levels simultaneously, exhibiting pH-responsive swelling. For this reason, in an aim to achieve all of the properties mentioned above, the process of crosslinking started to be viewed as a viable solution. It is well known that the crosslinking of chitosan significantly affects the swelling and sorption potentials, as well as its transport and mechanical properties.

In order to obtain crosslinked chitosan, different types of methods and agents can be applied. For this purpose, primarily chemical and physical modifications are used. In the case of physical methods, where ionic interactions between the polymer chains occur, among different inorganic ionic compounds, calcium chloride and pentasodium tripolyphosphate are applied as crosslinking agents [1,2,3].

**Figure 1:**
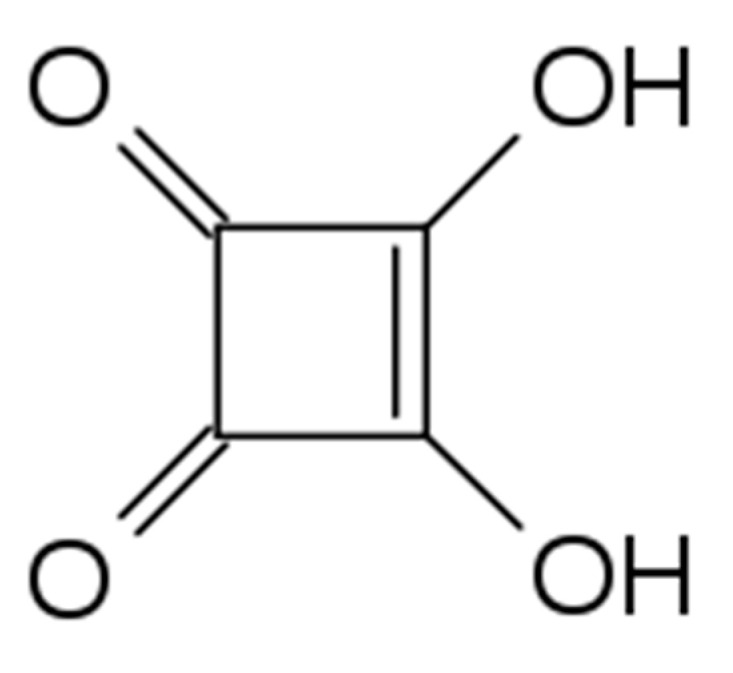
Chemical structure of squaric acid (H_2_SQ).

In the case of chemically crosslinked materials, the most popular crosslinking molecules include: glutaraldehyde [4,5], formaldehyde [6], boric acid [7], epichlorohydrin [8], and ethylene glycol diglycidyl ether [9]. These crosslinking agents, however, are characterized by toxic properties in their unreacted forms. For this reason, although a high degree of crosslinking can efficiently be achieved in their case, further application is significantly limited. A solution to the toxicity dilemma can be attained by application of a new crosslinking agent; for example, one of the aldehyde-functionalized compounds such as cellulose [10], β-cyclodextrin [11], starch [12], dialdehyde starch [13], or dextran [14]. Moreover, instead of toxic crosslinking agents, enzymatic crosslinking by means of transglutaminase can be applied [14,15,16,17]. Despite the considerable number of components used as crosslinking agents, new reactants are still being sought and tested. One of the components which can be used as a chitosan crosslinking agent is 3,4-dihydroxy-3-cyclobutene-1,2-dione, known as squaric acid (H_2_SQ). It should be stressed that squaric acid is a cyclic organic acid without any carboxyl groups (Figure 1). Its name refers to the square shape of the molecule created by four interconnected carbon atoms. The reactivity of squaric acid with amine groups was presented in the work of Wurm and Klock [18]. The formulation of magnetic nanoparticles coated with chitosan and crosslinked with squaric acid was described by Ziegler-Borowska et al. [19]. Moreover, squaric acid was described by Skopińska-Wiśniewska as an effective crosslinking agent of the collagen/elastin hydrogels [20]. It is worth noting that Skopińska-Wiśniewska et al. [20] analyzed also the biological properties of collagen/elastin materials crosslinked by H_2_SQ. The in vitro test performed with the 3T3 cell line revealed that the addition of the crosslinking agent resulted in only a slight, not statistically significant, decrease in the cellular response. Based on these findings, it can be suggested that H_2_SQ can be regarded as a safe compound in comparison to other, toxic crosslinking agents indicated above.

Obtained results allowed us to establish that squaric acid readily reacts with both of the protein materials mentioned above. To the best of our knowledge there is no indication of any paper reporting on the effect of squaric acid as the crosslinking agent of chitosan-based films.

For this reason, in the present work, the influence the introduction of squaric acid into the chitosan matrix has on its properties has been examined.

In order to establish the extent of changes occurring in the structure as well as in the morphology after an addition of squaric acid, Fourier transform infrared spectroscopy (FTIR) and atomic force microscopy (AFM) were applied. The thermal stability of chitosan before and after crosslinking was analyzed. Moreover, mechanical properties, as well as the swelling of the obtained materials in mediums characterized by different pH levels, were determined. Chitosan crosslinked by means of squaric acid presented significantly improved mechanical and swelling properties. The obtained results clearly show that squaric acid belongs to the group of effective crosslinking agents.

## 2. Results and Discussion

### 2.1. Fourier Transform Infrared Spectroscopy

**Figure 2:**
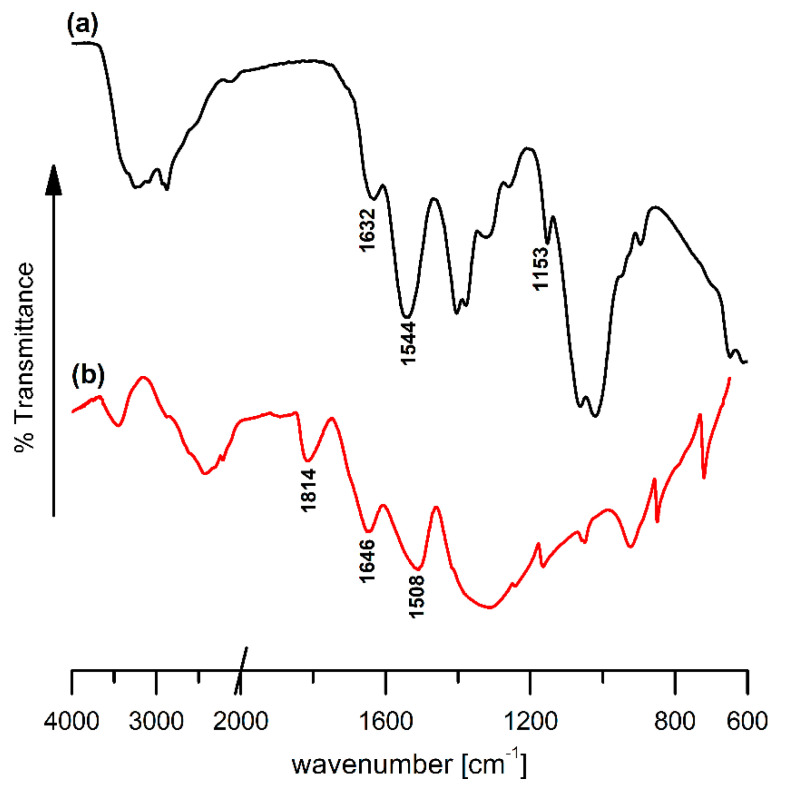
(**a**) Fourier transform infrared—Attenuated Total Reflectance (FTIR-ATR) spectrum of chitosan (Ch) and (**b**) Fourier transform infrared (FTIR) spectrum of squaric acid (H_2_SQ).

**Figure 3:**
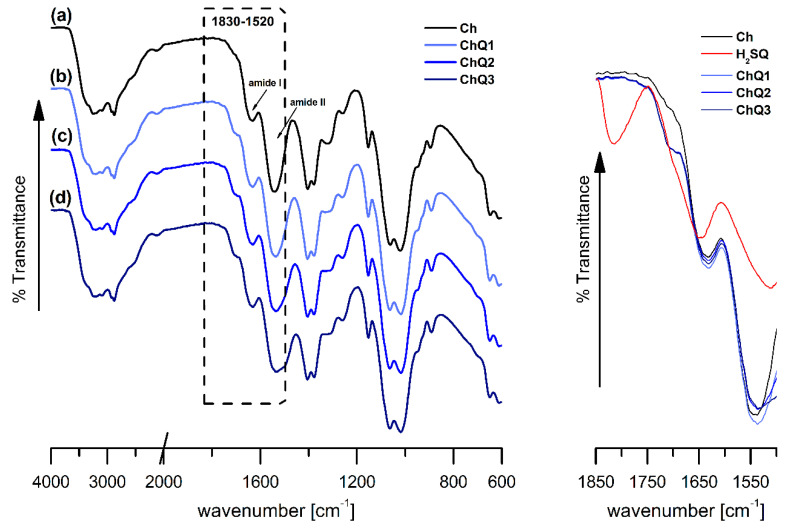
FTIR-ATR spectra of (**a**) chitosan (Ch) and squaric acid-crosslinked chitosan, (**b**) ChQ1, (**c**) ChQ2, and (**d**) ChQ3 (left: stacked spectra, right: overlapped spectra).

To confirm the internal chemical structure of the chitosan-based films and to prove the successful chitosan crosslinking with 3,4-dihydroxy-3-cyclobutene-1,2-dione (squaric acid, H_2_SQ), the FTIR spectrum of the H_2_SQ in KBr-disc form, along with the Fourier transform infrared—Attenuated Total Reflectance (FTIR-ATR) spectra of pristine chitosan, and H_2_SQ-modified chitosan films were compared (Figure 2 and Figure 3).

The spectrum of neat chitosan film (Figure 2a) is consistent with the spectra described earlier in the literature [21,22,23,24]. Among numerous vibrational bands typical of chitosan and indicated previously [21,22,23,24], the most characteristic include: ν(O–H) and ν(N–H) stretching vibrations (maximum at 3251 cm^−1^), ν(C=O) stretching in the amide group (amide I vibration, 1632 cm^−1^), δ(N–H) bending in the amide group (amide II vibration, 1544 cm^−1^), antisymmetric stretching of the C–O–C bridge (1153 cm^−1^), and skeletal vibrations involving the C–O stretching (1062 and 1021 cm^−1^).

The infrared spectrum of the squaric acid (Figure 2b) shows a strong broad band at 1508 cm^−1^ representing overlapped stretching ν(C=C) and ν(C=O) vibrations [25,26]. The presence of a C=O bond can be also confirmed through the asymmetric stretching vibration ν_as_(C=O) at 1814 cm^−1^. A very intense band at 1314 cm^−1^ represents the stretching ν(C–C) vibrations. Other, less intense bands correspond to [25,26,27]: ν(C=C) stretching (1646 cm^−1^), ν(C–C) stretching (1166, 1049, 924, and 850 cm^−1^), and ring breathing (C–C in-phase (symmetric motion) ring stretching vibrations, 721 cm^−1^).

A comparison of FTIR spectra of the pristine chitosan film (Ch), squaric acid, and H_2_SQ modified chitosan films (ChQ1, ChQ2, and ChQ3, see Materials and Methods) (Figure 2 and Figure 3) revealed differences in the regions corresponding to C=O and -NH_2_ vibrations (1830–1520 cm^−1^ region). After adding the crosslinking agent, absorption bands related to the N–H bending in the amide group (amide II vibration) changed in their positions, while C=O stretching in the amide group (amide I vibration) was almost unaffected (Table 1). The peak at 1544 cm^−1^ shifted to ca. 1536 cm^−1^ and in the region of about 1720–1560 cm^−1^ a broad band with a maximum at approximately 1631 cm^−1^ and a small shoulder at ca. 1700 cm^−1^ was observed.

According to our previous findings [2,28], and based on the knowledge that Schiff bases absorb in the range 1620–1660 cm^−1^ [29], it is reasonable to assume that the intense band at ca. 1631 cm^−1^ is a result of overlapping of the amide I vibration of chitosan and the C=N stretching of a Schiff base. As can be seen, the intensity of this band increases slightly after squaric acid crosslinking (Figure 3 (right)), in comparison to the neat chitosan film spectrum. Similar observations have been made by Ziegler-Borowska et al. [30] in relation to biopolymeric magnetic nanoparticles crosslinked with glutaraldehyde and squaric acid, who also indicated that the increase in the band intensity was the result of the formation of new C=N bonds. Moreover, Souza et al. [31] also suggested, in the case of H_2_SQ-modified chitosan, that a new band at 1536 cm^−1^ should be assigned to the ν(C=N). As indicated by Ziegler-Borowska et al. [30], in the spectra of chitosan/squaric acid/Fe_3_O_4_ systems a band at 1438 cm^−1^ was noted and assigned to the -OH (-O-) bending vibrations from squaric moiety. The band was not noted in the ChQ spectra (Figure 3b–d, left) as it corresponded to a similar wavelength as the C–H vibration at CH_2_ groups in the pyranose ring of chitosan (Figure 3a, left).

**Figure 4:**
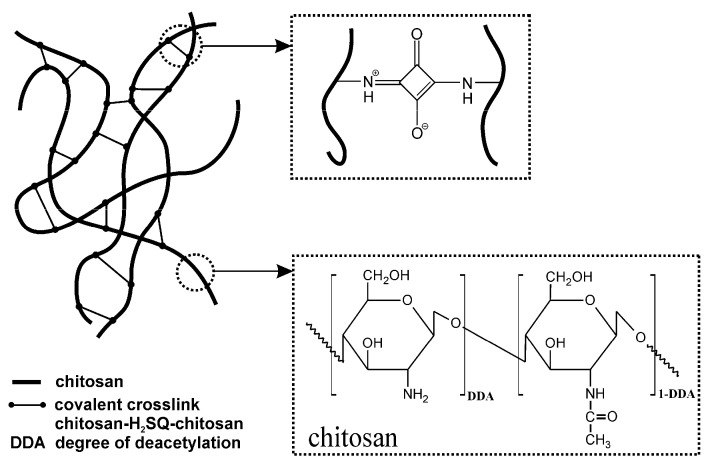
Schematic chemical structure of chitosan crosslinked with squaric acid.

The changes discussed above indicate that the formation of chemical crosslinks between chitosan and squaric acid, schematically shown in Figure 4, has indeed taken place.

Analysis of the crosslinked structure presented in Figure 4 suggests the presence of C=O functional groups in the ChQ structure. It can also be noticed, however, that in the spectra of ChQ the band characteristic of squaric acid at 1814 cm^−1^ (ν_as_(C=O)) is missing. The lack of this band was also observed in the H_2_SQ-crosslinked chitosan spectrum presented by Souza et al. [31], despite the fact that this sample was obtained with an excess of the crosslinker (polymer:crosslinker weight ratio = 1:2). It is worth noting that the amount of the crosslinking agent (squaric acid) in ChQ1–ChQ3 films is relatively small when compared to the mass of the crosslinked polymer (up to 3 wt.% of chitosan), thus changes observed in FTIR spectra are also relatively small and bands’ intensities are dominated by the vibrations of functional groups present in chitosan. Based on the known value of the chitosan degree of deacetylation, and the relative amounts of chitosan and the crosslinker, assuming that one mole of the crosslinker reacts with two moles of the -NH_2_ functional groups (Figure 4), the theoretical degree of chitosan crosslinking with squaric acid was found to equal 4.19, 8.38, and 12.57% in the case of the ChQ1, ChQ2, and ChQ3 samples, respectively.

### 2.2. Swelling

One of the most important features of neat chitosan and chitosan-based materials is their swelling behavior in contact with different external media. It is a crucial property in terms of evaluating possible biomedical and pharmaceutical applications, especially in controlled drug delivery systems, and in the food packaging industry, as it determines the diffusion of solute, wettability, and mechanical properties.

According to the Flory–Rehner theory, in general, a crosslinked polymer gel immersed in an external media and in the process of reaching an equilibrium with its surroundings, is subject only to two opposing forces, i.e. the thermodynamic force of mixing and the retractive force of the polymer chains [32,33]. These forces become equal at equilibrium state, thus the physical situation can be described by the following Gibbs free energy formula:

(1) $$\Delta G_{total}=\Delta G_{elastic}+\Delta G_{mixing},$$

where $\Delta G_{elastic}$ represents the elastic retractive forces inside the material and correlates with the degree of crosslinking, while $\Delta G_{mixing}$ represents the mixing of the solute molecules with the polymer chains and can be regarded as an affinity between the polymer and the components of the external fluid (polymer–solvent interaction).

Theoretical considerations about equilibrium swelling become more complex for those polymeric systems in which ionic moieties are present. Due to the ionic nature of the polymer network, additional contribution ($\Delta G_{ionic}$) must be taken into account in the total change in Gibbs free energy [32,33]:

(2) $$\Delta G_{total}=\Delta G_{elastic}+\Delta G_{mixing}+\Delta G_{ionic}.$$

**Figure 5:**
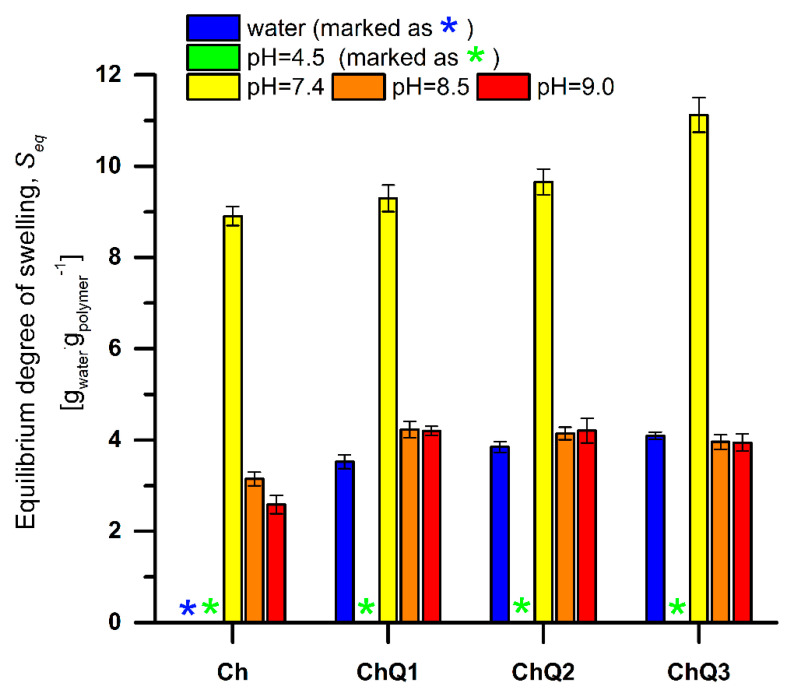
Equilibrium swelling degree of non-modified and squaric acid-crosslinked chitosan films in water and different buffered solutions.

As it was already proved by other researchers [34,35], polymeric materials formed from weak polyelectrolytes, like chitosan, undergo changes in swelling in response to changes in pH and ionic strength. In many cases, in evaluations of the effect of different pH levels on equilibrium swelling value, the ionic composition of the buffer is neglected. In Figure 5 the equilibrium swelling values of Ch and ChQ films in pure water and different buffered solutions of constant ionic strength are compared.

The swelling capacity of all Ch and covalently crosslinked chitosan (ChQ) films is highly dependent on the pH of the swelling medium. As can be seen, both non-crosslinked and crosslinked chitosan films undergo dissolution in an acidic (pH = 4.5) media. The $S_{eq}$ value decreases with increasing pH; however, differences between pH 8.5 and 9.0 solutions, for ChQ films, are within the measurement error.

**Figure 6:**
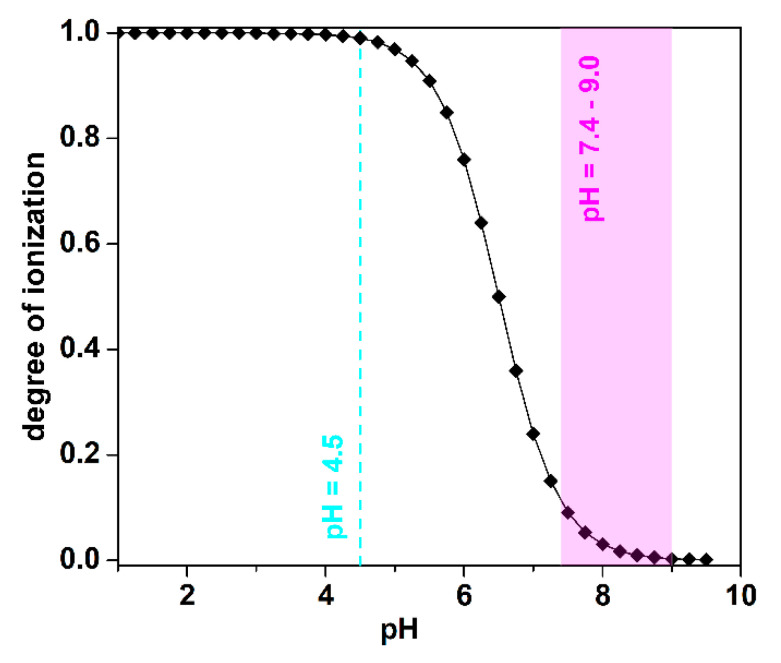
Degree of ionization of chitosan vs. pH.

The disintegration of all of the tested films occurring in pH 4.5 results from the chemical nature of chitosan and the relatively small degree of crosslinking (the highest: 12.57% degree of crosslinking for ChQ3). Chitosan belongs to the weak polybases and is characterized by pKa ≈ 6.5 [36]. At pH < pKa the -NH_2_ functional groups of Ch are almost completely ionized, forming -NH_3_^+^ (degree of chitosan ionization 99%, Figure 6), increasing simultaneously with the osmotic pressure inside the polymeric network. Repulsion of the equally charged -NH_3_^+^ functional groups also enhances the relaxation of macromolecular chains. As a result, in these pH conditions, the water uptake is so high that it leads to mechanical disintegration. Comparison between Ch and ChQ films indicates that the number of formed chemical crosslinks, as well as their strength, and the 12.57% degree of crosslinking, are not high enough to prevent breakdown.

Data presented in Figure 5 confirm that all, non-crosslinked and crosslinked, chitosan films swell, do not disintegrate, and reach equilibrium swelling state in buffered solutions of pH higher than 7.4. The differences in swelling capacity with an increasing pH correspond to the chemical nature of the polymeric network and confirm that one of the major factors contributing to the swelling of chitosan-based materials is the state of ionizable functional groups present within the network. At pH higher than 7.4 only a few amino functional groups are ionized (degree of chitosan ionization < 10%, Figure 6), thus repulsive forces are lower than those in the polymeric films swelled at pH 4.5. As a result of an increasing pH, a decrease in $S_{eq}$ is observed and the films’ swelling is mainly controlled by hydrophilic groups of all components.

The observed differences in swelling of the squaric acid-crosslinked chitosan films are also related to the number and nature of interactions between the chitosan chains and the crosslinking agents. Generally, a lower crosslinking density results in a higher swelling capacity [33,35]. In turn, a higher crosslinking density causes a simultaneous reduction in the degree of swelling values and the pH-sensitive swelling, while improving the stability of the network [37,38]. In the crosslinked networks, however, the hydrophilic/hydrophobic balance and tacticity should also be taken into account [39]. Analysis of the $S_{eq}$ values for the Ch, ChQ1, ChQ2, and ChQ3 (Figure 5) films indicates that covalent crosslinking with squaric acid slightly increases the swelling of the Ch materials, even if the degree of chitosan crosslinking increases. As confirmed during the FTIR analysis, the ChQ1, ChQ2, and ChQ3 films differ in the number of covalent crosslinks, with the highest count recorded in the case of the ChQ3 material. In general, the presence of chemical crosslinks reduces the chain mobility and also reduces swelling. Higher $S_{eq}$ values of the H_2_SQ-crosslinked chitosan networks at pH > 7.4 (in comparison to neat Ch) indicate that the hydrophobic/hydrophilic balance plays a crucial role in swelling properties. Neat chitosan films are regarded as hydrophilic due to the presence of amine (-NH_2_), hydroxyl (-OH), and ether (C-O-C) polar groups, that can be involved in interactions with water through the hydrogen bonds. Even if the crosslinking process reduces the number of free amino groups, the –NH– functionals are still present (Figure 4). Moreover, the new functional groups C=O and C-O- appear. Shikata and Okuzono [40], who determined the hydration numbers of compounds containing a carbonyl group as equal to ca. 0 at 25 °C, classified these compounds as the newly defined “hydroneutral” substances. Such hydroneutral compounds display very weak interaction with water molecules due to their unique characteristic of zero hydration number. The C=O functional groups, present in a crosslinked chitosan network, can act as hydrogen bond acceptors, enhancing the interactions between the polymeric network and water, and finally enhancing swelling ability. A similar effect on the swelling capacity, resulting from the presence of the C=O functionals, was observed earlier by Ewing et. al [41], in the case of the CO_2_ sorption by different polymeric materials (unsaturated polyketones). These authors concluded that the concentration of the Lewis basic groups in polymers increased the overall polymer swelling. In summary, both differences in crosslinking density and in hydrophilicity of the network affect the equilibrium swelling value. Crosslinking with H_2_SQ decreases swelling capacity, while the presence of the C=O functionals derived from the crosslinker enhances this property. As for ChQ films swelled at pH 7.4, the equilibrium swelling value increases with an increasing crosslinker amount, which leads to the conclusion that the effect of the crosslinker–water interactions prevails over the effect of a higher crosslinking density.

A similar phenomenon is noted in the case of H_2_SQ-crosslinked chitosan samples swelled in water (Figure 5). The highest value of equilibrium swelling degree (4.09 ± 0.08 g_water_·g_polymer_^−1^) is observed in relation to the ChQ3 sample, representing the highest degree of crosslinking value. It should also be emphasized that, contrary to the pristine chitosan film, all crosslinked chitosan samples do not disintegrate in H_2_O media. This clearly indicates that the crosslinking process, and newly covalent crosslinks formed within the chitosan material, improve the chitosan film stability. Comparison of swelling data for neat and crosslinked chitosan in water (pH = 7, Figure 5, blue bar) and in buffered solution of pH = 7.4 (Figure 5, yellow bar) confirms the conclusion that ionic strength and the presence of ionic species in the solution also affect swelling of polyelectrolyte-based materials.

### 2.3. Changes in Surface Morphology Determined Using AFM Technique

**Figure 7:**
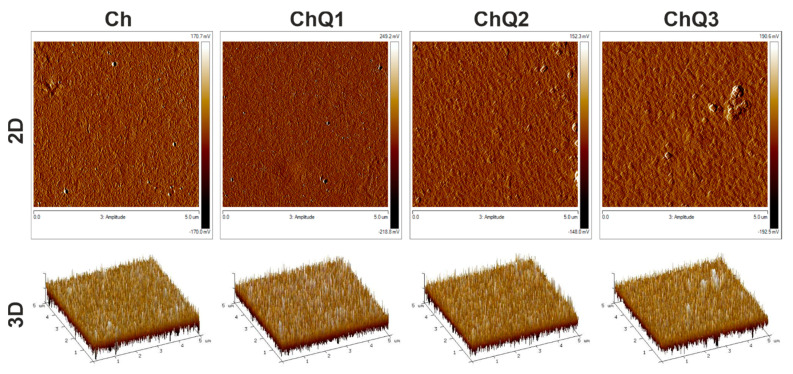
2D and 3D atomic force microscopy (AFM) images of the surface topography of Ch and ChQ films.

The 2D and 3D surface morphology images of films obtained from pure chitosan and chitosan crosslinked by adding 1, 2, and 3 wt.% of squaric acid are presented in Figure 7. The corresponding roughness parameters are shown in Table 2, where the R_q_ and R_a_ values are presented. R_q_ (root mean square roughness) represents the standard deviation of the distribution of surface heights. R_a_ (arithmetic average height parameter) is defined as the average absolute deviation of the roughness irregularities from the mean line over one sampling length [42]. It is the most universally recognized roughness parameter estimate used in quality control.

To determine the impact of squaric acid addition on chitosan films, pure chitosan film was used as a control sample. In particular, the influence of the amount of squaric acid on surface parameters was tested. As it can be seen (Figure 7), the AFM images do not reveal differences in the surface properties of films in the case of a pure chitosan film compared to crosslinked films. Two-dimensional images indicate the homogeneity of each tested sample consisting of chitosan and squaric acid. An imperfection was observed in the 2D image of chitosan with the 3 wt.% addition of squaric acid (Figure 7: ChQ3). This can either result from a contamination of the sample or suggest that chitosan and squaric acid solutions are not always uniformly miscible.

The slight differences are visible in the values of roughness parameters. The surface roughness increases with an increasing content of the crosslinking agent (Table 2). This may indicate an increase in the heterogeneity of crosslinked samples in comparison to neat chitosan film. The surface roughness parameters’ values, both R_q_ and R_a_, are highest in the case of chitosan crosslinked by 3 wt.% of squaric acid (3.67 and 2.95 nm, respectively). The observed changes in morphology are related to the interactions between polymeric compounds (chitosan) and the crosslinking agent (squaric acid). Neat chitosan films exhibit the lowest R_a_ and R_q_ values which most likely occurs due to the high intrinsic chain stiffness of this polymer [43]. Addition of the crosslinker into the film-forming solution, and formation of additional covalent crosslinks between polymeric chains causes a reduction in chain mobility. Consequently, after drying, a slightly less uniform, rougher surface is observed (Table 2). A higher content of the crosslinking agent in chitosan-based films and a higher number of covalent crosslinks results in a slight increase in the values of the R_q_ and R_a_ parameters. The effect of other crosslinking agents on chitosan and its mixtures was observed earlier by other researchers. In the work of Sionkowska et al., dialdehyde starch was a modifier for chitosan and mixtures of collagen, hyaluronic acid, and chitosan [44]. After modification, the observed roughness was higher for crosslinked materials than in the case of pure chitosan, but lower for crosslinked polymeric mixtures than uncrosslinked ones [44]. In relation to chitosan and chitosan/montmorillonite materials crosslinked by glutaraldehyde, roughness parameters’ values were lower after modifications [3]. In the case of chitosan films crosslinked with tannic acid, a similar correlation to the one in our research was observed [45]. Roughness parameters’ values were higher for chitosan with 5 and 20% of tannic acid content, as the crosslinking agent. To the best of our knowledge, there is no information on the influence of squaric acid on polymeric films’ roughness.

### 2.4. Thermal Properties

**Figure 8:**
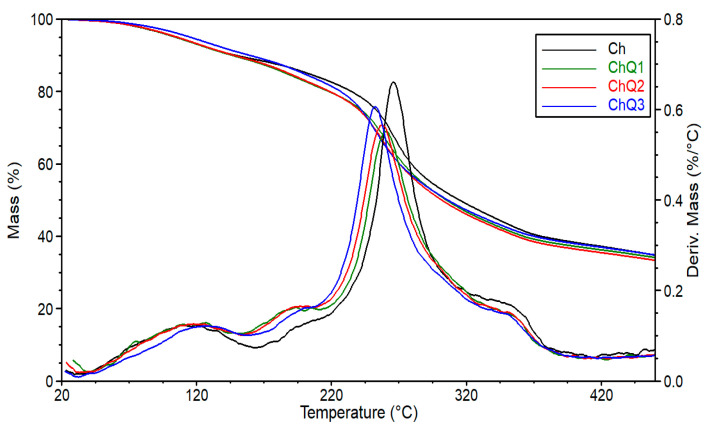
TG and DTG curves of unmodified (Ch) and modified chitosan (ChQ).

Thermogravimetric analysis is one of the techniques often used to establish the influence of different additives on the thermal stability of polymeric-based materials, a crucial feature of its further application in different areas. For this reason, the effect of squaric acid on the changes in thermal properties of the investigated materials, consisting of chitosan and squaric acid, have been analyzed. Figure 8 shows TG (thermogravimetry) and DTG (differential thermogravimetry) curves obtained in the case of unmodified chitosan and chitosan crosslinked using a different amount of squaric acid. The values of temperatures at 5 and 10% mass loss (T_5%_, T_10%_) of the chitosan-based materials were selected and presented in Table 2.

In the case of samples crosslinked by means of 1 and 2 wt.% of squaric acid no noticeable change in the values of the analyzed parameters are observed. This is consistent with the literature, where glutaraldehyde was applied as the crosslinking agent of chitosan [3]. It is well known that chitosan-based films are formed from a water solution and mass lost up to 10 wt.% occurring in a range between 50 and 130 °C can be mostly attributed to moisture vaporization [46]. The second stage of decomposition observed in Figure 8 (140–265 °C) is related to the degradation of chitosan [47].

However, taking into account the obtained results, it has to be noted that the addition of 3 wt.% of a crosslinking agent causes a significant increase in thermal stability of the obtained material. This suggests that squaric acid in higher concentrations is able to enhance intermolecular interaction and simultaneously stimulate hydrogen bond formation. Therefore, it is justified to assume that this leads to the formation of a more compact molecular structure of the polysaccharide carbon chains which are less susceptible to decomposition [48]. To summarize, the analysis of the thermal properties and stability indicates that a high amount of squaric acid can significantly affect the thermal stability of chitosan-based materials.

### 2.5. Mechanical Properties

**Figure 9:**
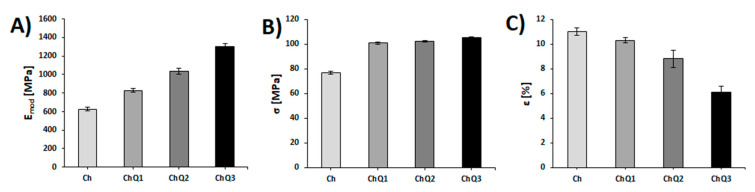
(**A**) Young’s modulus (E_mod_); (**B**) tensile strength (σ), and (**C**) percentage elongation (ɛ) of chitosan and chitosan with squaric acid 1, 2, and 3 wt.% addition.

To establish the influence of squaric acid on the properties of chitosan-based films, the mechanical resistance was tested. Mechanical parameters such as Young’s modulus (E_mod_), tensile strength (σ) and percentage elongation at break (ɛ) in relation to chitosan and chitosan with 1, 2 and 3 wt.% squaric acid addition, have been determined and compared. The results of Young’s modulus, tensile strength, and percentage elongation are shown in Figure 9.

The addition of squaric acid increases the Young’s modulus’ values, which is indicative of the elasticity and rigidity of the sample [49]. It should be stressed that the increase in the values of the Young’s modulus, in comparison to pure chitosan, is observed in the case of all of the obtained materials with an addition of squaric acid. Moreover, it can be seen that a film made of chitosan with a 3 wt.% addition of squaric acid is characterized by the highest values of the Young’s modulus. The introduction of a crosslinking agent into chitosan causes the films to become less flexible and more rigid than a pure chitosan film. An analogous correlation has been noted in the case of collagen/elastin blends crosslinked by squaric acid [20]. The value of E_mod_ calculated for the ChQ3 sample (1302±34 MPa) is more than twice as high as the value recorded in relation to chitosan before modification (625 ± 21 MPa). In the work of Skopińska-Wiśniewska [20], the same tendency has been described: a high-compression modulus in relation to all of the crosslinked collagen–elastin based samples.

The obtained results lead also to the conclusion that films with an addition of squaric acid exhibit higher values of tensile strength (σ) than films made of chitosan without crosslinking (Figure 9B). This clearly indicates that the force which was applied to break the crosslinked films was greater than the force required to break the samples without the crosslinker. After the crosslinking process, the tensile strength increases. The greatest tensile strength value was observed in the case of chitosan with 3 wt.% addition of squaric acid. Wegrzynowska-Drzymalska et al. studied chitosan films crosslinked with dialdehyde starch (5 and 10 wt.%), dialdehyde chitosan (5, 10, and 15 wt.%), and glutaraldehyde [50], and also noted that tensile strength increased with an increased amount of the crosslinker in the chitosan films. They reported that the most significant differences in tensile strength values were observed in relation to chitosan crosslinked with dialdehyde chitosan. Similar observations were made by Tang et al. [51] in the case of dialdehyde starch-crosslinked chitosan films. The authors reported that the tensile strength of all of the crosslinked films (1, 3, 5, 7, and 9 wt.% of crosslinker) were higher than that of the pure chitosan film and increased with the increase in the dialdehyde starch content.

The studied materials are characterized by the value of percentage elongation at break (ε) inversely proportional to the value of the Young’s modulus (Figure 9C). When the Young’s modulus’ value is higher, the percentage elongation value is lower, with the sample being more rigid and less elastic. This phenomenon can be attributed to the reduced mobility of polymeric chains related to the crosslinking process. The addition of H_2_SQ results in the disruption of the existing hydrogen bonds in chitosan, while new strong covalent crosslinks are formed at the same time. A similar effect of crosslinking on material elasticity was observed by Frick et al. [52]. Wegrzynowska-Drzymalska et al. [50] indicated that the elongation at break of chitosan-based materials decreases when a higher amount of crosslinking agent (glutaraldehyde) is used. Pavoni et al. [53] also investigated the effect that addition of glutaraldehyde (0.5, 1, 2, 5, and 10 wt.% glutaraldehyde content) had on the mechanical properties of chitosan. They observed that the application of the crosslinking agent promoted a decrease in the elongation at break, from 45±3% (pure chitosan) to 3±1% in the case of chitosan crosslinked with 10 wt.% of glutaraldehyde. All of the findings discussed above are consistent with those presented in Figure 9 relating to H_2_SQ-crosslinked chitosan.

These observations lead to the conclusion that increasing the dose of a crosslinking agent is effective in inducing significant changes to the mechanical properties of the films. The addition of squaric acid to chitosan has an effect on all of the mechanical parameters of the films measured in this study. This suggests that during the addition of squaric acid to chitosan, crosslinking reactions in the film occur and are responsible for an improvement in mechanical properties. All of the above discussed changes in E_mod_, σ, and ɛ parameters stay also in agreement with the results of FTIR analysis: a higher content of crosslinking agent in chitosan-based films results in a higher number of covalent crosslinks, thus a more rigid polymeric matrix is formed.

Various crosslinking agents have been tested recently, e.g., glutaraldehyde, formaldehyde, dialdehyde starch, tannic acid, and others [51,54,55,56]. Many of these compounds can effectively be used in crosslinking polymers. Researchers, however, are constantly looking for new crosslinking agents to improve the properties of polymers. Squaric acid, due to the presence of functional groups, is very reactive and therefore the mechanical properties of the modified materials are superior to the ones displayed by pure chitosan [57].

### 2.6. Differences in Color of Chitosan-Based Materials

It is well known that color psychology plays an important role, especially in advertising and retail sales. A significant number of consumers decide to buy a particular product only based on the product’s color, particularly when the products are homogeneous. For this reason, color is often taken into account by material designers and psychologists. Bearing in mind how important coloristics are, different methods are used to establish the most appropriate shade and color of materials. One of the popular techniques is the CIE Lab system. This method was also applied in the case of other materials, which were the object of our previous studies [58]. Based on the values of the measured parameters L, a, and b, the ∆E parameter was calculated (Table 3).

The obtained results clearly indicate that the amount of squaric acid used as a crosslinking agent significantly influences the final color of the obtained materials in comparison with unmodified chitosan. Based on the results presented in Table 3 it is apparent that the ∆E values of ChQ films exceeds 20. Taking into account that significant changes in color can be noticed by a consumer when the value of ΔE exceeds 5, the obtained results lead to the conclusion that the application of squaric acid will be clearly visible to potential consumers. The tests reveal that the most significant changes were observed in the case of the L and b parameters. The increase in b parameter indicates that the color of materials is a more intense yellow in comparison with pure chitosan, while a decrease in the L parameter value suggests that chitosan crosslinked using squaric acid tends to be darker when compared to the original sample.

## 3. Materials and Methods

### 3.1. Materials

The commercially available chitosan from crab shells of 72.25 ± 0.77% degree of deacetylation was supplied by BioLog Heppe GmbH (Landsberg, Germany). Squaric acid (H_2_SQ, Sigma-Aldrich, Germany) was applied as the crosslinking agent. Acetic acid was purchased from Avantor Performance Materials Poland S.A. (Gliwice, Poland). To perform the analysis of swelling the following buffer solutions of constant ionic strength were prepared: acetic buffer (pH = 4.5) and Tris buffers (pH = 7.4; 8.5; 9.0). The reagents purchased at Avantor Performance Materials Poland S.A. (Gliwice, Poland) used for the preparation of the buffer solutions mentioned above were as follows: hydrochloric acid 35–38%, sodium acetate, sodium chloride, Tris(hydroxsymetyl) aminometane, and sodium chloride.

### 3.2. Formation of Chitosan-Based Films

In order to obtain uncrosslinked and crosslinked chitosan-based films, the casting and the solvent evaporation techniques were used. In the first stage, the 1 wt.% chitosan (Ch) solution was prepared by dissolving chitosan in 2% (*w*/*v*) acetic acid. In the second stage, squaric acid, dissolved in a small amount of distilled water, was introduced into the chitosan solution. The squaric acid weight equaled 1, 2, or 3 wt.% of the chitosan mass. All of the obtained solutions were cast on polystyrene plates and dried for 48 h at 37 °C. Subsequently, plates were placed in vacuum for 24 h at the same temperature. It needs to be noted that the ratio between the solution volume and the polystyrene plate surface was adjusted to eventually achieve a 0.1 mm film thickness. The obtained pure chitosan and chitosan-based films containing 1, 2, and 3 wt.% of the crosslinking agent were described as: Ch, ChQ1, ChQ2, and ChQ3 respectively.

### 3.3. Methods of Analysis

#### 3.3.1. Fourier Transform Infrared Spectroscopy (FTIR)

The Fourier transform infrared—Attenuated Total Reflectance (FTIR-ATR) spectra, obtained using zinc selenide crystal, of Ch and ChQ films were registered within the frequency range of 500–4000 cm^−1^. The FTIR spectra of squaric acid (H_2_SQ) were recorded in KBr disc form using a device working in transmittance mode. Final spectra are a result of 64 scans at the spectral resolution of 2 cm^−1^. In each of the (FTIR and FTIR-ATR) analyses Nicolet iS10 (Thermo Fisher Scientific, Waltham, USA) was applied. OMNIC 7.0 software (Thermo Fisher Scientific, Waltham, USA) was also employed for spectra analysis.

#### 3.3.2. Atomic Force Microscopy (AFM)

The topographic images were obtained using a multimode scanning probe microscope with a NanoScope IIIa controller (Digital Instruments, Santa Barbara, CA, USA) operating in the tapping mode, in air, at room temperature. Surface images were acquired at a fixed resolution (512 × 512 data points) using a scan width of 5 μm with a scan rate of 1.97 Hz. Three replicates were performed in the case of each film. Silicon tips with a spring constant of 2–10 N/m were used. Parameters such as the root mean square roughness (R_q_) and roughness average (R_a_) were calculated from 25 μm^2^ surface area using NanoScope Analysis software.

#### 3.3.3. Thermogravimetric Analysis (TG)

Simultaneous TGA-DTA Thermal Analysis type SDT 2960 (TA Instruments, New Castle, DE, USA) equipment was applied in order to determine the thermal stability of the obtained materials. All measurements were performed under air flow from room temperature to 600 °C at a heating rate of 10 °C/min. Two replicates were performed in the case of each film.

#### 3.3.4. Mechanical Properties

**Figure 10:**
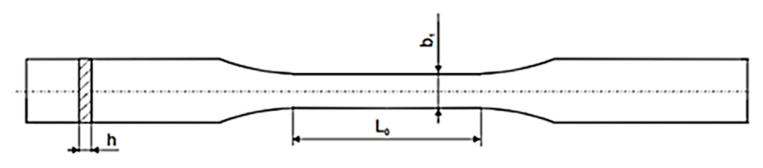
Shape of samples with dimension markings.

The mechanical properties of the materials were measured using a mechanical testing machine: TIRAtest 27025. Young’s modulus, tensile strength, and percentage elongation of polymer films based on chitosan with an addition of squaric acid were measured at room temperature in the dry state and at a crosshead speed of 50 mm/min in accordance with the procedure outlined in the PN-EN ISO 527-1-3 standard. Samples were cut into paddle-shaped pieces, with initial dimensions that constituted 25 mm in length (L_0_), 5 mm in width (b_1_), and 1 mm thickness (h) (Figure 10). The thickness of the samples was measured by a type A-91 ultrameter (producer: Manufacture of Electronic Devices, Warsaw, Poland). All of the film samples were cut using the same shaper. In the case of each type of film, at least five samples were tested.

#### 3.3.5. Color Measurement

The color of chitosan films undergoing squaric acid crosslinking was determined using a MICRO-COLOR II LCM 6 (Dr Lange) colorimeter. Based on the CIE Lab system the color difference (ΔE) of materials was calculated. In order to establish the total values of ΔE the Equation (3) presented below was applied.

(3) $$\Delta E=\sqrt{{\left(L-L^{*}\right)}^{2}+{\left(a-a^{*}\right)}^{2}+{\left(b-b^{*}\right)}^{2}},$$

Particular characteristics were described using the following parameters: L—lightness, a—colors ranging from green (−a) to red (+a), and b—colors ranging from blue (−b) to yellow (+b). The color of the studied materials was expressed as L (lightness), a (redness/greenness), and b (yellowness/blueness) values [58]. Chitosan film without an addition of squaric acid was used as a reference sample ($L^{*}$, $a^{*}$, and $b^{*}$ values). Three replicates were performed in the case of each film.

#### 3.3.6. Swelling

The swelling behavior of pristine chitosan and squaric acid-crosslinked chitosan films was evaluated with the gravimetric method. Dry polymeric film of 1 cm × 1 cm was weighed ($W_{0}$, ca. 20 mg), placed in 100 cm^3^ of swelling media (T = 37.0 ± 0.1 °C), and kept for 24 h under gentle stirring. The 24 h swelling time was determined, through the swelling kinetic measurements, to be sufficient to reach an equilibrium swelling state by all tested films. The swollen film was removed from the solution and weighed ($W_{eq}$) after an excess of solvent was eliminated with filter paper. The equilibrium degree of swelling ($S_{eq}$ [g_water_·g_polymer_^−1^]) was calculated as:

(4) $$S_{eq}=\frac{W_{eq}-W_{0}\;}{W_{0}}.$$

To avoid an effect of pretreatment on the swelling of the polymeric film, samples of similar thickness and surface, dried in analogous conditions, were used. Final $S_{eq}$ values constitute a mean from three independent measurements. In each case relative standard deviations were lower than 5%.

In all swelling experiments distilled water and the following buffered solutions of constant ionic strength (0.145 M) were used: 10 mM acetic acid/sodium acetate solutions (acetate buffer, pH 4.5) and 10 mM Tris buffered solutions (pH 7.4, 8.5, and 9.0).

#### 3.3.7. Theoretical Degree of Chitosan Crosslinking

The theoretical degree of crosslinking [%] was calculated by the amino content of chitosan after H_2_SQ addition and the amino content of raw chitosan:

(5) $$Degree\;of\;crosslinking=\frac{I_{NH2}-F_{NH2}\;}{I_{NH2}}\cdot 100\%,$$

where $I_{NH2}$ and $F_{NH2}$ are the initial and final moles of NH_2_ functional groups in chitosan samples, respectively. The difference $I_{NH2}-F_{NH2}$ represents the moles of NH_2_ groups involved in the formation of the crosslinks, assuming that one mole of the crosslinker reacts with two moles of the -NH_2_ functional groups, and can be calculated as:

(6) $$I_{NH2}-F_{NH2}=2\cdot \frac{m}{M},$$

where *M* is the molar mass of squaric acid, 114.06 g·mol^−1^, and *m* represents the H_2_SQ mass per known amount of crosslinked chitosan, equal to 0.01, 0.02, and 0.03g per 1g of chitosan, for ChQ1, ChQ2, and ChQ3 samples, respectively.

## 4. Conclusions

Novel crosslinked materials based on chitosan and squaric acid were successfully formed. Their structure and morphology were established by means of FTIR and AFM techniques. The analysis of the FTIR spectra confirmed that the covalently crosslinked chitosan films were obtained. The other fact which must be noted is that the addition of squaric acid did not significantly influence the roughness of the obtained chitosan-based films. Because of the apparent compatibility, the surface of the crosslinked materials was smooth.

The introduction of squaric acid into chitosan significantly improved Young’s modulus and tensile strength. The same tendency was observed in the case of the thermal stability of the crosslinked materials. These changes are a direct result of the crosslinks formed between chitosan and squaric acid. Evaluation of the color changes showed that squaric acid can play an important role in the perception of the obtained materials by prospective consumers.

Simultaneously, it was also proved that all films being in contact with different external media differ in swelling capacity as a result of different pH levels affecting the ionization of functional groups, crosslinking density, and hydrophobic/hydrophilic balance. All films were stable in a solution of pH higher than 7.4. Moreover, crosslinked chitosan samples did not dissolve in water and as little as 1 wt.% of the crosslinker ensured the stability of the chitosan films in this media.

In conclusion, based on the performed analyses, it was established that squaric acid is an efficient crosslinker that can effectively replace currently used, toxic crosslinkers like glutaraldehyde.

## Figures and Tables

**Table 1:** The position of characteristic bands [cm^−1^] in the FTIR spectra of H_2_SQ powder, and Ch- and squaric acid-modified chitosan films.

Sample	C=O	C=C	Amide I	Amide II
H_2_SQ	1814	1645	- ^2^	- ^2^
Ch	- ^2^	- ^2^	1632	1544
ChQ1	- ^2^	~1700 ^1^	1631	1537 ^3^
ChQ2	- ^2^	~1691 ^1^	1631	1536 ^3^
ChQ3	- ^2^	~1700 ^1^	1631	1536 ^3^

^1^ visible as a small shoulder on the amide I vibration band. ^2^ not detected. ^3^ amide II and C=N.

**Table 2:** Values of roughness root mean square roughness (R_q_) and roughness average (R_a_) parameters and thermal stability of studied materials.

Sample	R_q_ [nm]	R_a_ [nm]	T_5%_ [°C]	T_10%_ [°C]
Ch	2.30 ± 0.02	1.89 ± 0.03	104.9 ± 0.2	145.7 ± 0.6
ChQ1	2.68 ± 0.04	2.19 ± 0.10	105.4 ± 0.2	147.2 ± 0.3
ChQ2	2.99 ± 0.04	2.51 ± 0.11	106.2 ± 0.4	147.9 ± 0.2
ChQ3	3.67 ± 0.05	2.95 ± 0.13	115.8 ± 0.5	158.6 ± 0.9

**Table 3:** Color variables of studied materials.

Sample	Color Variable ^1^			
	L	a	b	ΔE
Ch	90.40 ± 0.23	−1.40 ± 0.02	5.04 ± 0.02	-
ChQ1	82.51 ± 0.13	−3.21 ± 0.06	24.39 ± 0.09	21.0 ± 0.19
ChQ2	79.94 ± 0.09	−3.93 ± 0.06	27.63 ± 0.11	25.0 ± 0.21
ChQ3	77.42 ± 0.21	−4.23 ± 0.09	29.51 ± 0.07	27.9 ± 0.13

^1^ mean value (three replicates).

## Data Availability

The data presented in this study are available on request from the corresponding author.
